# Book Reviews

**Published:** 1806-06

**Authors:** 


### CRITICAL ANALYSIS
OF THE
### RECENT PUBLICATIONS
ON THE
### DIFFERENT BRANCHES OF PHYSIC, SURGERY, AND MEDICAL PHILOSOPHY.

---

*Notes on the West Indies, &c. including Obſervations on the Iſland of Barbadoes, &c. &c. with occaſional Hints regarding the Seaſoning, or Yellow Fever of Hot Climates.* By G. Pinckard, M. D. Deputy Inſpector-General of Hoſpitals to His Majeſty's Forces, 3 vols. 8vo pp. about 360 each. London: 1806.

This intereſting and entertaining work would not have fallen within the notice of our department, had it not been for the latter part of the title page. We obſerve, however, that independent of yellow fever, many valuable obſervations, intimately connected with the ſcience and practice of medicine, are occaſionally introduced, particularly in the two laſt volumes.

Of ſome of theſe we ſhall give an account in our future numbers; at preſent, we ſelect the following deſcription of the *Elephantiaſis* of *Barbadoes*, or the *Bardadoes diſeaſe*.

" It commonly appears in the form of an enormous and frightful enlargement of one or both legs; but occaſionally affects other parts, particularly the ſcrotum, which becomes increaſed to a ſurprizing bulk. When once eſtabliſhed, it is extremely difficult to remove, and for the moſt part proves to be incurable. It affects the general health, leſs than might be expected, and frequently exiſts for many years without ſeeming materially to impair the conſtitution; often, indeed, the perſon attacked with it bears it about throughout the remainder of a long life. It is moſtly ſeen among the negroes, but it is too common alſo among the creole whites, and even ſuffers not the Europeans to eſcape. Although ſo frequent in Barbadoes, as to be held in a great degree peculiar or endemial, it is not wholly confined to this country: ſome inſtances of it being ſeen in the neigbouring iſlands.

" It would ſeem not to have been ſo prevalent as it now is from any very diſtant period of time; for about the year 1760 died at Barbadoes a man named Francis Briggs, more commonly known by the fictitious appellation of Chriſtopher Columbus, who, from the uncommon and monſtrous appearance of his legs, had been repreſented as the bug-bear or object of terror for the purpoſe of frightening children.

" Male and female, young, middle-aged, and old, black and white, are now all ſubject to its attack; and, in walking the ſtreets, the eye is diſtreſſed, at almoſt every corner, with the appearance of this hideous deformity.

" The

" The difeafe ufually begins with an affection of the inguinal glands, from whence a red ftreak, or line of inflammation extends down the limb, in the direction of the lymphatic veffels; the part affected becoming tumefied, and taking on a fhining and œdematous appearance. The fwelling gradually occupies the whole of the leg, increafing until, in many inftances, the limb is more than double its ordinary fize. The fkin affumes a morbid appearance, grows rough and fcaly, or is covered with irregular wart-like rifings. In fome cafes deep belts or indentations appear in various parts of the tumor, as if formed by the preffure of ligatures: in others the fwelling bulges out in a number of irregular protrufions: fometimes, from extreme diftention, the fkin ruptures or breaks into cracks and fiffures, and a watery fluid oozes out, which on expofure to the air grows gelatinous upon the furface. The foot frequently partakes of the difeafe: but in many cafes the immenfe tumor of the leg terminates abruptly at the ancle, hanging over the foot in knotty and fcaly excrefcences. The deformity thus becomes diverfified—the enormous bulk of leg appearing under a variety of unfeemly and difgufting forms. As the enlargement increafes, the whole extremity becomes hard and fcaly; and the diftended fkin, which, at firft, indented, grows thick and corneous, and wholly refifts the preffure of the finger.

" It has been found on diffection that, from the effufed lymph which originally caufed the tumor having become coagulated and hardened, the fubftance of the enlarged limb has affumed an appearance not unlike brawn—the morbid fkin, and the cellular membrane under it, having grown into a tough, horny, and almoft cartilaginous confiftence.

" From this unfightly malady being moftly accompanied with fever of an intermittent type, we often hear it termed " the fever and ague." Indeed, from the periodical returns of the paroxyfms, and from the tumefaction fucceeding to them, the difeafe has been very generally confidered only as an effect refulting from intermittent fever. The practice, faid to be fuccefsful in removing it, feems alfo to be founded upon this view of it. Regard being had to the fever as the original affection, the elephantiafis is confidered only as a fequel, and the curative means are directed folely to the removal of the febrile fymptoms: which being effected by antimony and the bark, the patient is fent for a time to fome other ifland, by way of change of climate, in order to prevent a relapfe. No particular attention is paid to the tumor, which, on the fever being removed, is expected gradually to fubfide. But fometimes, inftead of receding, it remains ftationary, or is increafed; or if it did fubfide, is renewed on any future recurrence of the fever.

" Often a return to Barbadoes brings a return of the intermittent, and a confequent addition to the enlargement of the already thickened extremity; and from the attacks of the difeafe recurring in frequent repetition, there remains no way of preventing it from being eftablifhed into an unfeemly deformity, but by feeking the remedy of a more temperate climate. Frequently the diforder feems to be entirely fubdued by a few years refidence in England, yet again recurs on the patient returning to Barbadoes.

"Except on its early attack, or at the periods of acute relapfe, the difeafe is attended with little pain, and the enlargement fometimes proceeds fo gradually, as for the perfon himfelf to be in a degree infenfible of it. He walks about as ufual, and appears to fuffer but little inconvenience, either from the additional bulk, or the great increafe of weight. Hence it is often lefs afflicting to the individual, than offenfive to others. It is extremely repugnant to the fight; and as the negroes go about the ftreets with thefe difeafed limbs expofed to every eye, Europeans, but recently arrived, are extremely annoyed by their filthy and monftrous appearance.

"Perhaps nature has not formed, nor can the human mind conceive an object at once fo difgufting, and fo pitiable, as an old half-famifhed negro woman—of withered frame—tottering and trembling about with her loofe and naked fkin hanging fhrivelled in deep furrowed wrinkles; and dragging after her one or both legs grown into an immenfe bulk of hideous difeafe—her feet only toes, protruding from this huge mafs of diftempered leg. Yet fuch are the objects too often feen hobbling about the ftreets of Bridge-town!" [ To be continued. ]

---

### *Memoirs of the Medical Society of London;*

[ Continued from page 377—386. ]

"Article 16.—*Case of a Wound in the Peroneal Artery, in which the Limb was saved by removing a Portion of the Fibula.* By Thomas Croxall Cam, Surgeon, Bath, late Senior Surgeon to the Infirmary at Hereford."

"Article 17.—*Observations on the Medical Use of the white Oxyd of Bismuth.* By Alex. Marcet, M. D. &c. Sec. M. S. one of the Physicians to Guy's Hospital."

This remedy was suggested to the writer of the Paper during his stay at Geneva by Dr. Odier, Professor of Physic in that place. The following is the manner in which it is prepared:

"The magistery of bismuth is prepared by dissolving a quantity of very pure bismuth in nitric acid, and precipitating it by water, or by a solution of potash. But if the bismuth is not very pure, if for instance it is mixed with nickel, the precipitate is not perfectly white; it is then mixed with a greenish precipitate, which is nothing but an oxyd of nickel which water will not precipitate; for which reason we are more certain of obtaining a pure precipitate of bismuth by water than by potash.

"I use this remedy with success in doses of six grains, four times a day, in all cases of spasms of the stomach, brought on by any kind of aliment, and proceeding only from the irritability of that organ. This complaint is extremely frequent at Geneva, particularly particularly amongst servant maids who are in the habit of carrying water on their heads, and make great use of their arms.

"I have published my observations on this subject in two papers, one of which has been printed in the "Journal de Medicine," and in the "Journal Encyclopedique" of Paris for the year 1796; and the other, sent by Dr. Belcombe to the Royal Society of Gottingen, has been printed in a German journal from whence Dr. Murray has drawn, in his "Apparatus Medicaminum," a very detailed extract."

"Dr. Odier, in addition to this account, assured me repeatedly that he had tried the oxyd of bismuth in much larger doses, and that he had never observed it to produce any bad effects whatever; whilst, on the contrary, he had hardly ever known it to fail in effecting a cure, when used in the circumstances that have just been mentioned."

The first instance in which Dr. Marcet tried this remedy, was one of those formidable cases of dyspepsia, in which, nothing is taken into the stomach without producing a disposition to vomit, followed by the rejection of the greater part of the food. The common stomachic remedies were first tried without effect.

"On the 21st the symptoms being exactly the same, the castor oil was repeated, and a mixture of magnesia, rhubarb and peppermint-water, was substituted for the bitter infusion. I did not hasten to try any of the metallic medicines, because I conceived her complaint to depend, either upon some organic disease within her stomach, or upon some spasmodic affection of that organ; and I did not imagine, that, on the former supposition, any of the metallic medicines could be of material service; whilst, on the latter, I wished first to give a fair trial to the bismuth, a supply of which I expected to be able to procure in a few days.

"On the 30th, the symptoms continued as before, but she said the mixture gave her a momentary ease, and could sit on her stomach longer than any thing else. The same medicine was ordered to be repeated, and some of the *pilulæ aloes cum myrrha* were directed to be taken occasionally.

"On the 2d of August, there was little or no change in the symptoms. Some drops, consisting of æther, tincture of bark and vitriolic acid, were added to the former medicines.

"On the 17th, she complained of not deriving any sensible benefit from her medicines; she then looked thinner than when I had seen her first, but not so much as might have been expected from the nature of her complaint; yet her general character and appearance, and still more so, her perseverance, and the regularity with which she came for advice, at stated periods, from a great distance, did not permit me to suspect any deceit or misrepresentation in the account which she gave of her complaint. On that day, I ordered five grains of the oxyd of bismuth, with fifteen grains of the pulv. tragacanth. compos. to be taken three times a-day.

"On

"On the 24th she came to me with an uncommonly animated countenance, saying she was almost quite well; but she begged I would let her have some more of those powders that had produced such remarkably good effects; to which request I most readily consented.

"On the 31st she said she was quite free from complaint, but still requested I would allow her to take the powders a few days longer, as she was afraid of a relapse if she should leave them off all at once. She accordingly continued to take two of these powders a-day, for about a week longer; when finding herself perfectly free from complaint, she discontinued all kinds of medicines.

"Since that time I did not hear any more of this woman, until about the middle of November last, when she called upon me, not to complain of a relapse, as I first apprehended, but to thank me again for the blessing of health to which she had been so happily restored. She said, however, that although she was in general quite free from her complaint, yet if any thing *fretted her*, and particularly if her children gave her any uneasiness, she felt, occasionally, some slight sensation of it."

This medicine promises to be an important addition to our list of stomachics; and when we consider how very numerous the causes, and how dreary the consequences of dyspepsia are, we shall not doubt the necessity of recording every remedy, which may relieve so important an organ as the stomach. Several other cases are added; and the paper concludes with some observations on the necessity of accurately preparing the oxyd.

"Article 18.—*Of the Use of Bath Waters in Ischias, or Diseases of the Hip-Joint, commonly called a Hip-Case.* By W. FALCONER, M. D. F. R. S. &c.

This is a very valuable paper on a disease, the frequency and usually slow progress of which, render it surprising that so little has been said on the subject. Dr. Falconer traces it through all its varieties, and all its stages, with much accuracy and diligence. The result of his researches is, that in the early stages, a cure may be expected from a judicious use of the Bath Waters. In its more advanced stage, great relief, if not a permanent cure; but that whenever the disease has advanced to suppuration, especially if hectic symptoms have come on, that the remedy much exasperates the disease. The only objection we meet with to any part of the paper, is, the apprehension the author expresses, lest the matter should be absorbed after being deposited. That the Bath waters may be likely to increase the hectic, we have every reason to suppose, from what we see in common phthisis; but we thought, by this time, that the idea of hectic being caused by the absorption of matter, had been long since exploded.

The description of the disease is followed by an account of the manner of treatment which the author has found most effectual in addition to the Bath Waters, with a Table of the success attending these combined remedies; and the whole is followed by a very a very learned analysis of what has been done by his predecessors, beginning with Hippocrates and finishing with Mr. Foord. In the first part of this history, Dr. Falconer stands on much firmer ground than we gave him credit for in his opinions concerning the nature of morbus cardiacus. In both, indeed, he evinces great learning; but in the dissertation now before us, he shows a judgment equal to his learning.

As this disease is a source of permanent misery to many, and as its slow progress enables most of the sufferers to arrest it in time, we shall, in order to increase the publicity of the relief experienced by the Bath Waters, extract the Table given by the author, and his explanatory remarks.

"*TABLE of the State of the Patients at their Discharge, who were admitted into the Bath Hospital for Hip-cases, from May* 1*st*, 1785 *to April* 7, 1801, *classed according to their Ages.*

| Ages | Cured | Much better | Better | No better | Improper | Irregular | Dead | Total |
|---|---|---|---|---|---|---|---|---|
| Under 10 years | | 5 | 8 | 1 | 9 | | | 23 |
| From 10 to 20 | 30 | 24 | 32 | 9 | 34 | | 2 | 131 |
| From 20 to 30 | 20 | 48 | 28 | 13 | 34 | 2 | 1 | 146 |
| From 30 to 40 | 22 | 29 | 18 | 2 | 24 | 3 | | 98 |
| From 40 to 50 | 21 | 30 | 15 | 7 | 16 | 3 | | 92 |
| From 50 to 60 | 8 | 25 | 6 | 1 | 5 | 2 | 2. one of the ſmall-pox. | 4 |
| From 60 upwards | 2 | 7 | 4 | | | 3 | 1. of the ſmall-pox. | 17 |
| Total | 103 | 168 | 111 | 33 | 122 | 13 | 6 | 556 |

"It is proper to apprize the reader that by *cured*, in the second column of the foregoing table, is meant such persons who have completely recovered from their complaint, and who have *no* symptoms of the disease remaining, for which they were admitted. By *much better* is understood such as have nearly recovered, but have have still some stiffness, debility, or other mark of the disease remaining. This term, however, is never applied, unless to such as are nearly recovered, and never to crippled or helpless persons, however such may be circumstanced with regard to health.

"By *better* we understand persons who have received obvious and material advantage, but who have nevertheless strong marks of the effects of the disease. This term, however, is never applied to such as, although they may have received some temporary alleviation of their sufferings, still labour under hectical or other symptoms, that indicate their health to be declining.

"It is much to the credit of the Bath hospital, that a great degree of candour has been uninterruptedly preserved ever since its foundation, above 60 years ago, in representing the state of the patients when dismissed. These, when minuted to be discharged by the attending physician, are again produced before some of the other professional persons, and examined as to the state they were in when admitted, which is compared with their state when examined, and both these are compared with the report of their state by the attending physician. They are again produced before the committee, and separately and regularly examined as to the same points, and I have repeatedly witnessed the committee requesting the attendant physician, to alter the report, when it appeared to them that the amendment was more considerable than it was put down in the report, but I never knew the smallest hint offered, that the state of the patient was more favourably represented by the physician, than it seemed to merit, on the examination before the committee. In short, it has been the invariable rule to err, if at all, rather on the side of caution, than on the contrary extreme, and to represent such patients only to have received benefit in any degree, whose cases exhibited obvious and undeniable marks of amendment, not such as are merely probable, or any-wise equivocal.

"It appears from the foregoing report of the state of the patients, that out of 556 persons admitted into the Bath hospital for hip-cases from May 1, 1785, to April 7, 1801—103, or about 1. in 5.398 received a complete cure, that 168 or 1. in 3.03095 received great benefit, and were nearly recovered; that 111, or nearly one-fifth of the whole received some benefit, and that the aggregate of these three numbers, amounting in the whole to 382, or as 1. in 1.4555, or more than two-thirds, received advantage from a trial of the remedy. Of the above numbers, four only died in the hospital of the disease, a very inconsiderable proportion, 33 or nearly a 17th part of the whole were no better, 122 were deemed improper cases for a trial of the waters, and 13 were discharged for irregularity.

"By those set down under the title *improper* are meant, in general, such whose cases were, on their first examination, or soon after it, thought to be improper subjects for a trial of the waters, as being in too advanced a stage of the disease, or from other other circumstances of their health that forbad the use of the remedy; much the greatest part of whom ought not to have been sent hither at all. In 97 of these out of 122, matter was discovered to be formed, or forming, very soon after their arrival, which of course rendered a trial of the waters inadmissible. These, therefore, should be struck out of the account, as proving nothing respecting the efficacy or inefficacy of the waters. The same, it is obvious, may be said of the 13, who were discharged for irregularity, and indeed of those who died, as four of these were, when sent, not in a condition to be removed with safety and propriety, and two died of the small-pox.—This takes off 141 from the list, and reduces the whole number, that should be considered on this occasion, to 415. The proportions then will stand thus.

"Cured 1—in 4.1553 nearly.

"Much better 1—in 2.54, or nearly two-fifths.

"Better 1—in 3.74.

"Proportion of those who received benefit to the whole number as—9.2048.—to 10. or above nine-tenths of the whole.

"It it unnecessary to observe how much the foregoing calculations, which are taken from the register of the hospital, a most accurate and authentic medical record, are in favour of the efficacy of the Bath waters in hip-cases, and it should *be noticed* that they plead strongly for a trial of them in the early stages of the disease. It is more than probable that a large proportion of the unsuccessful cases, amounting in the whole to 159, including those who were *no better—improper*—and those who *died* of the disease, would have received relief, had a timely application been made to this remedy.

"Very few of those specified as improper were suffered to make any trial of the waters, and in 97 of them, as I have before observed, matter was discovered at their arrival, or soon after, and the hectical symptoms precluded all hopes from the use of the bath, and indeed left little from the trial of any other means.

"It appears that the Bath waters applied in an early stage of the disease have been nearly equally successful at very different ages. Their good effects have been manifested as early as five years old, and as late as 70 years, and the proportion of those who received relief at 60 years old and upwards, was as large as in the early periods of life.

"The average stay in the hospital, of the first thirty of the persons *cured*, is 105 days, of the same number of those who were discharged *much better* 155 days, and of the same number of those who were discharged *better* 138 days nearly. The average of the stay of those who were benefited is nearly 133 days, or 19 weeks.

"It appears that the Bath waters are more successful in hipcases cases at a warm time of the year than at a cold one, as is indeed the case with this remedy, when applied to other disorders.

" Of 88 persons taken in order, who received benefit, and who were admitted in the months of April, May, June, and July, 25 were *cured*, 39 were *much better*, and 24 *better*.

" Of 105 persons received in October, November, December, and January, 25 were *cured*, 41 were *much better*, and 38 *better*.

" It is obvious that a larger proportion of those who were admitted in the spring and summer, and who had a prospect of a series of warm weather, received a greater degree of benefit than those who were admitted in the autumn and winter.

" I have thus finished my remarks on the tables, and trust I have established the efficacy of the Bath waters in this obstinate, painful, and dangerous disease.

" They are undoubtedly very effectual, but much time is usually necessary to complete a cure; and indeed, it needs be no cause of surprise, that a disease should take up as many months in its cure, as in some instances it has lasted years before the remedy was applied. In very recent cases I have seen a few weeks complete a cure."

" Article 19.—*Observations on the Position of Patients in the Operation of Lithotomy.* By NATH. SMITH, M. D. &c. New Hampshire."

Dr. Smith had occasion to perform the operation on a man seventy-two years of age, whose limbs were so inflexible by previous rheumatic affections, that his thighs could not be separated more than six inches, and his knees scarcely be bent. His lower limbs were therefore supported at a right angle from his body; his hips raised higher than his head; and his superior extremities unembarrassed. By this posture, Dr. Smith conceives, the operation, may, in some subjects, be performed with greater ease, the abdomen not being brought into an unnatural position. This paper contains some useful hints, and probably, the causes assigned by the author, may, sometimes, prove the impediment to the removal of the stone. At all events, it may be right under any difficulties to see what effect a greater relaxation of the bandages may have on the form of the bladder, and the state of the abdominal muscles.

" Article 20. *Case of Enlargement of the Scrotum.* By RIGBY BROADBENT, M. D. &c. Of Spanish Town, Jamaica."

" Article 21.—*Two Cases of Diabetes, with Observations on the different States of that Disease.* By JOHN BOSTOCK, M. D. &c. Liverpool."

The name of the author is enough to impress our readers with a due opinion of the value of this paper. The experiments are minute, as they ought to be, and doubtless not less accurate. From the nature of the communication it is impossible to compress it.

The succeeding article consists of questions on the Influenza, contained in a circular letter from the Medical Society to its corresponding Members. This is followed by forty-three answers, called as many articles, and occupying considerably more than half the book. As those who undertook to read and print all this, have not taken the trouble of making any arrangement, it will not be expected that we should take that charge on ourselves.

" Article 80.—*Further Account of the lithontriptic Power of the muriatic Acid, as observed by* Mr. Peter Copland, *in Lithiasis, and Icterus calculosus.*—Extracted from a Paper, dated Swayfield, December, 1800."

This valuable communication the Society have thought it right to shorten, the facts contained in it serving only to confirm the contents of a paper from the same Gentleman published in a former volume. We shall only transcribe, for the use of our readers, the manner in which the remedy was exhibited.

" The dose of acid used by Mr. Copland is from 30 to 50 drops, three times a-day. The constant and uniform effect of this medicine, after a few doses, is stated to be the appearance of a considerable quantity of calculous sediment in the urine. This sediment is described as consisting, in almost every instance, of *red sand*, the nature of which does not appear to have been chemically examined by the author. In a few cases, however, there seems to have been also a discharge of light-coloured calculous matter; and in one instance, two ash-coloured concretions, weighing six grains, were found in the urine.

" The general consequence of this discharge appeared to be a considerable relief of all the symptoms, and, in by far the greatest number of the cases related, a permanent cure was supposed to have been effected. In no instance, however, was the presence of a stone in the bladder ascertained by examination previous to the exhibition of the acid; but from the quantity of calculous sediment obtained by the use of this medicine, amounting in one case to 110 grains, and in several to nearly as much, no doubt can be entertained as to the nature of the complaint. As a proof that this deposition was not afforded immediately from the urine, the author relates a case, supposed to be calculous, in which eleven ounces of the acid were taken, in doses of 60 drops, three times a-day, without producing the least sediment in the urine; and it was afterwards proved that the complaint had been mistaken, and that there had been no stone in the bladder.

" After the author's great success with the acid in lithiasis, he was led to try its effects in Icterus calculosus, and relates three cases in which he has every reason to suppose that this medicine had the desired effect; but confesses that the event of so few cases is not sufficient to enable him to draw any positive conclusion with regard to the last disease, although he considers it as well worth the attention of practitioners."

" Article

"Article 81.—*Sketch of a new Theory of Cow-pock, with Remarks on contagious Disorders.* By JAMES SIMS, M. D. &c. &c. President of the Society.

The learned President begins this paper by a theory of animal and vegetable processes, deriving them all, or most of them, from fermentation. His position is, that there are different genera of fermentation. But, before we talk of genera, it behoves to define what we mean by the thing itself. — Dr. Sims not having told us what he means by fermentation, it is impossible we should undertake to confute or confirm his theory. If all that is meant by fermentation, is, that a body composed of various materials is to go through a certain process, after which it will be so far altered that it cannot go through the same again, we shall be very much at a loss to understand how fermentation can be the cause of vegetation; for admitting this fermentation to be excited by solar heat, we see the same process going on, if not from day to day, at least from season to season.

"Still carrying on," continues our author, "the chain of reasoning, do we not see in the animal, and particularly in the human frame, the progress of fermentation distinctly pointed out? A little of the matter of any of the contagious disorders is inserted into the body, and that disorder is produced and no other; and as in the fermentation of liquids, after the process has been gone through, it is impossible to excite the same kind of fermentation again in the same materials, even by the addition of a ferment, so, after the infectious disease has subsided, the frame becomes incapable of it again. Is not this a plain reason for our not being liable to the small-pox, and other contagious diseases, constitutionally, a second time?"

But here we should reflect, that when a decoction of malt is fermented, its change throughout is such, that it no longer consists of particles similarly arranged, or having similar properties: which is not the case with a man after and before the small-pox, for, unless he is marked with the disease, he may easily be mistaken for the same individual. It should further be remarked, that though we are in the habit of putting a small quantity of ferment into wort, in order to hasten its fermentation, yet, such an addition is by no means necessary, as the process will take place without it; whereas, we have reason to believe, small-pox, and are certain that cow-pox, will never happen to any man, unless some of the contagious matter is brought into contact with him.

The author proceeds:

"Whilst a student at College I maintained in a Medical Society, an opinion that all contagious eruptive diseases were communicated to mankind from other animals, among a particular species of which each particular disease might be said to be epizootic."

As Dr. Sims has, by his own account, practised forty years, this this opinion of his must be of an early date; however, the first regular surmise of the kind that we meet with in print, is contained in Mr. Hunter's Treatise on the Venereal Disease. It is probable the opinion may be older, as it is well known that some derive the name of syphilis from such a source; it is even probable that Mr. Hunter might have heard of cow-pox before the date of his book, as it is well known that Dr. Jenner's account was read before the Royal Society: but as that learned body did not think proper to publish it, we cannot ascertain whether the communication was prior or subsequent to Mr. Hunter's publication. After this, we find the same suggestion in Doctor Adams's Treatise on Morbid Poisons; he surmises the probability, that many of these diseases may have originated from such causes, and instances the cow-pox and hydrophobia as two cases in point.— Dr. Jenner, from whom only, by means of Mr. Cline, it is now well known that Dr. A. derived his information concerning cow-pox, introduces his discovery by similar remarks. The opinion was indeed a natural result of so great a discovery, when Doctor Jenner had found that the disease originating in a horse, produced a well marked morbid poison in the cow, which was uniform in its character when transferred from one to the other; and still more, that it might be transferred to man, and from man to man, retaining its uniform figure and laws. We have however, as yet, nothing to prove that the cow-pox originates in the small-pox of man. The experiment has been often tried, and always ineffectually.

Dr. Sims has since suggested (see our Journal, vol. xiv. p. 480) that the spurious cow-pox may be the offspring of chicken-pox. This may be the case; but the opinion, like the former, is built on conjecture.

These remarks are followed by an account of the result of some experiments, which, as they serve to contradict a suggestion at one time pretty prevalent, ought to be recorded.

"In 1764, when I began the practice of physic, my thoughts were greatly employed upon inoculation of the small-pox. The Suttonian method coming into vogue about that time, greatly arrested my attention; and as many points respecting it were kept secret, my mind was naturally much occupied with investigating the circumstances which gave it celebrity. Among other things which occurred to me, I imagined that a proper dilution of the virus before insertion, might tend to render the disease milder. With this view, I tried a number of fluids, mostly bland ones, but must acknowledge that I totally failed in my intention, the only difference which I could perceive being, that the certainty of infection was much lessened, whilst the disease was not obviously mitigated."

The rest of the volume consists of prize questions proposed by the Society; a prize question on the yellow fever, proposed by the king of Prussia; and the customary accounts of presents to the Society.

Though

Though it cannot be denied, that this volume contains many useful papers, we are forced to admit, that the account of influenza occupies by far too large a portion, especially when we consider, that for want of arrangement few men in business can avail themselves of its contents, however useful they may be.— Another remarkable defect in a volume of the bulk like the present, and which, from the nature of its contents, must become a book of reference, is the want of an Index. This and the last volume are the only two out ef five chargeable with such an omission, which we hope, in the next, will be made up by a general Index to the three.

---

### *Dr. Pemberton's Practical Treatise on various Diseases of the Abdominal Viscera.*

[ Concluded from our laſt Number, p. 483. ]

When our author comes to treat of the diſeaſes of the kidnies, he introduces, what we conſider as a very intereſting diſtinction between the effects of the diſeaſes of the organs which increaſe and support the fleſh and ſtrength of the body, and thoſe which tend to diminiſh and exhauſt it.

" It is not so difficult," ſays Dr. P. " to diſtinguiſh an affection of the kidneys from diſeaſes of the other parts ſurrounding them, as it is to diſtinguiſh it from the diſeaſes of the other parts of the urinary ſyſtem.

" The ſenſations are communicated ſo readily by ſympathy from one extremity to the other of this ſyſtem, that the ſame ſet of ſymptoms attend, wherever the derangement in the urinary organs may exiſt. For example, a ſtone in the bladder ſhall give all the ſenſations of a diſeaſe in the kidneys; and again, a calculus in the kidneys ſhall give all the ſenſations and symptoms of a diſeaſe in the bladder. This obſcurity will ſhew the great caution neceſſary in pronouncing on the diſorders of the urinary paſſages.

" Another circumſtance no leſs remarkable, and which too will point out the neceſſity of caution in the prognoſtic, is the little diſturbance, which great and even fatal diſeaſes in this ſyſtem, occaſion in the conſtitution. Large ſtones have been repeatedly found in the bladder, and extenſive ulcerations in the kidneys, without their having in the leaſt diminiſhed the bulk of the body, or ſhewn any ſymptom, during life, by which their exiſtence had been ſuſpected.

" When there are but obſcure ſymptoms, which denote that ſome morbid change is taking place within the abdomen, this circumſtance of undiminiſhed bulk might help materially to diſtinguiſh diſeaſes of the kidneys from any chronic affection of the meſenteric glands, where there is uniformly the greateſt degree of emaciation.

" A proneneſs in the body to waſte or not, as the ſame diſeaſe ſhall happen to be ſituated in this or that part, is in itſelf a circumſtance very remarkable; and as an attention to this proneneſs may help to lead us through the obſcurities, which too often attend internal complaints, it is a ſubject well worthy of conſideration.

"To aſſiſt us in this inquiry, it may be right to ſpecify a few examples, where the difference of the effect of diſeaſe on the bulk is moſt ſtriking. Let us take the two caſes of a diſeaſed ſtate of the meſenteric glands, and a diſeaſed, or ſcrophulous affection of the breaſt. In the former we ſhall find there is great emaciation; in the latter, none at all.—In an ulceration of the ſmall inteſtines, great emaciation takes place; in ſchirrus of the rectum, none—In a diſeaſe of the gall bladder, which is ſubſervient to the liver, the bulk of the body is rapidly diminiſhed; but in a diſeaſe of the urinary bladder, which is ſubſervient to the kidneys, ſcarcely any diminution of bulk is to be perceived.—In an abſceſs of the liver the body becomes much emaciated; but in an abſceſs of the kidneys the bulk is not diminiſhed.

"If we examine into the functions of thoſe parts, the diſeaſes of which do or do not occaſion emaciation, we may perhaps be led to the true cauſe of this difference of their effect on the bulk. In order, however, to underſtand more clearly how the functions of theſe parts bear relation to each other, it may be neceſſary to premiſe, that the glands of the body are divided into thoſe, which ſecrete a fluid from the blood, for the uſe of the ſyſtem, and thoſe which ſecrete a fluid to be diſcharged from it. The former may be termed glands of ſupply; the latter, glands of waſte.

"The ſmall inteſtines, in conſideration of the great number of abſorbents with which they are provided for the repair of the ſyſtem, may be conſidered as performing the office of glands of ſupply.

"The large inteſtines, on the contrary, may be conſidered as performing the office of glands of waſte; inaſmuch as they are furniſhed very ſcantily with abſorbents, and abundantly with a ſet of glands which ſecrete, or withdraw from the ſyſtem a fluid, which ſerves to lubricate the canal for the paſſage of the fæces, and which itſelf, together with theſe fæces, is deſtined to be diſcharged from the ſyſtem.

"I have often imagined that this mode of conſidering the ſubject might, in many caſes, aſſiſt us in approaching to the ſeat of a chronic diſorder, by deciding where the diſorder is *not* ſituated, and conſequently by contracting within narrower limits the difficulties of our reſearches.

"Thus the ſymptom exhibited by the patient, either in retaining his bulk, or in being emaciated, might ſerve as a diagnoſtic, according to my conception, for the purpoſe of deciding, whether the diſorder is ſeated in the glands of ſupply, or in the glands of waſte.

"The glands which ſecrete a fluid to be employed in the ſyſtem, as well as the glands of direct ſupply, may be conſidered the Liver, the Pancreas, the Meſenteric glands, perhaps the Stomach, and the ſmall Inteſtines: and the glands of waſte are the Kidneys, Breaſts, Exhalant Arteries, and the large Inteſtines.

"In

"In an abfcefs of the Liver, and an abfcefs of the Kidneys, both of which glands frequently run into fuppuration, without exhibiting any pain in the part affected, it feems impoffible to decide in what part of the fyftem the derangement manifefted in both thefe cafes by the Hectic fever is fituated.

"According to the foregoing idea, if Emaciation takes place, we might then determine that the diforder muft be fituated in a gland of fupply; and thus we fhould be led to decide, that the diforder was certainly *not* in the Kidneys, confequently we fhould be fecured from the danger of mifapplying our remedies upon a part which was not affected.

"The fame Hectic attends a chronic difeafe of the mefenteric glands, and of the fmall inteftines: and here likewife, if emaciation does *not* take place, we fhould decide that the diforder was not fituated in thefe parts, or in the liver.

"Now it is furely of confiderable importance to determine where the diforder is *not* found, that our enquiries may be folely directed to thofe parts in which it is to be found.

"If this pofition refpecting the bulk of the body, under difeafe, fhould be admitted as true, will it not afford a probability that the Spleen, whofe difeafes produce great emaciation, is a gland of fupply?

"What has been here advanced muft be confidered as applying to local difeafes unattended by pain, as pain will itfelf fometimes wafte the body, though fometimes it will not. Here too the wafting from pain feems to vary according to the part from which it proceeds. A ftone in the bladder of urine, or in the Kidneys, nearly ftopping the difcharge of urine, and occafioning the greateft pain, will not in the leaft affect the bulk; but a biliary ftone, under fimilar circumftances, will occafion great and rapid emaciation.

"We muft obferve, however, that the prefence of pain in a part would fufficiently point out the feat of the difeafe. It is only in chronical complaints, where the morbid changes are fo gradual as to produce no pain, that thefe obfervations will particularly apply: and it is in difficulties of this nature, that we ftand in need of every help to affift us in forming a right judgment."

The extended ufe of the term *gland,* in the foregoing paffages, has drawn upon the author fome criticifms from Dr. Stone, in his work on the Difeafes of the Stomach, juft publifhed. Dr. S. objects to the ufe of the word Gland, when applied to the lungs, inteftines, &c.; but this apparent want of accuracy in terms cannot miflead the reader; nor does it, in our opinion, tend to invalidate the general inference. Thefe fhort fpecimens will enable our readers to judge of the originality and fpirit of inquiry, which pervades every part of this truly practical Treatife.

We are convinced that it will be read with great fatisfaction by the experienced practitioner, and with great improvement by junior ftudents. Nor can we doubt that it will rank as a medical claffic, the reading of which will become an indifpenfible obligation upon every ftudent of that profeffion.

*A Practical Treatise on the Diseases of the Stomach, and of Digestion; including the History and Treatment of those affections of the Liver and digestive Organs, which occur in persons who return from the East and West Indies; with Observations on various Medicines, and particularly on the improper use of Emetics.* By A. D. Stone, M. D. Fellow of the College of Physicians. 8vo. pp. 300. London: 1806.

It will strike our readers on comparing the title page of Dr. Pemberton's Work on the Diseases of the Abdominal Vicera and that before us, that the object of both authors, as far as appertains to the stomach and liver, is the same. This is by no means the case. In treating of the liver, Dr. P. confines himself principally to the acute and chronic inflammations of that organ and its hydatids. Our author, on the contrary, enters into the effects of hot climates and hard drinking, on the liver and stomach. On the diseases of the stomach Dr. P. employs only about 30 pages, whereas almost the whole of Dr. Stone's Work is devoted to the Anatomy, Physiology, and Diseases, of the organs of digestion.

In the former work, the symptoms and the cure are placed together; in the latter, both are treated at much greater length, and separately. Dr. S. also introduces the consideration of many diseases which are not mentioned by Dr. P.; as poisons, hypochondriasis, sick head-ache, chlorosis, tabes, and cutaneous diseases depending on the derangement of the stomach.

The 3d part of our author's work, in which he treats of the means of curing the diseases of the stomach, &c. the selection and the employment of remedies, we think particularly interesting in a practical point of view. And as we have received great satisfaction in perusing these two works of Dr. P. and Dr. S. jointly in the order we have noticed them, and as we think they mutually tend to illustrate each other, we recommend the same comparative view of them to our readers. As a specimen of Dr. S's style and manner of treating his subjects, we shall select his observations on the action of mercury, and the best mode of introducing it into the system. In the preceding chapter he had delivered the treatment of the stomach and abdominal viscera after residence in hot climates. The great importance of mercury had there been largely insisted upon, and the internal administration of it fully explained. In the present chapter, he delivers the treatment of that state of the stomach and abdominal viscera, which is induced by hard drinking.

"To introduce mercury into the system, so as to act powerfully on the liver, is the object which medical men attempt to accomplish in cases of this kind. Now, since mercury cannot produce its effect on the liver in any other manner, than by the change of action in the blood-vessels and absorbents consequent upon its stimulus, it is to be considered how far all or any of these actions are affectsd by it;—and this consideration is the more important from its having been stated by Dr. Saunders, Dr. Pemberton and others, that that mercury should be rubbed on the hypochondria, in preference to any other part, only because the mere friction upon the liver may be useful.

"The hepatic artery being very small, comparatively with the size of the liver, and as there is no possible reason to suppose that this artery is more affected by mercury, in any case of diseased liver, or in any other disease, than each individual artery in the body, there is little reason to suppose that any great effect can be produced on the liver from any alteration in the action of this artery, consequent upon the use of mercury.

"The influence of mercury on the veins has not at any time been proved to be considerable:—on the contrary, from all anatomical and physiological observation, it appears when taken into the blood-vessels to increase secretion generally, and that the power of any given quantity of mercury is exhausted in its operation on the salivary and excretory glands:——nor is there any reason, from any observation made on its action on the vena portarum, to suppose that its action on this vein, is greater than what it induces in any other vein in the body.

"It follows then, that the action of mercury on the blood-vessels of the liver, is proportionally to the size of this viscus, less considerable than on almost any other part of the body.

"The effect of mercury in producing absorption is obvious to the most superficial observation:—this effect probably depends, in some measure, on the necessity for absorption to supply the blood-vessels in compensation for the waste occasioned by the evacuant effects of mercury. When the liver is enlarged, as in some cases of persons, who have resided in the East and West-Indies (§ 40), when it is tuberculated, or when it is schirrous, as from the effects of spirituous liquors, the absorption of the adventitious or altered substance in the organic construction of the part, is the indication which principally demands attention, and from the known effect of friction in promoting absorption, it is reasonable from the benefit to be expected from friction alone, that mercury should be rubbed on the epigastric and hypochondriac regions rather than on any other part:—there is however the best ground for believing, that mercury, taken into the stomach or rubbed upon the skin near the liver, produces a much more immediate effect on the hepatic absorbents, and that these modes of its application are consequently, for this reason, especially, by far the best.

"Further it is to be observed, that the absorbents of the stomach and those conveying the chyle, often called the lacteals, anastomose freely with those from the liver, and therefore that mercury taken inwardly, will, when absorbed, immediately pass through and stimulate the absorbents coming out of the liver:—Mr. Cruikshank states in his second edition that he 'saw them filled with chyle from 'the mesentery, and which had passed through the substance of 'the liver, to the number of three hundred or more:'—As this observation must be considered to be correct, it not only strengthens ens the position here made, but it greatly elucidates the operation of alkohol in producing schirrus of the liver, which, as has already been mentioned (§ 42), is but ill explained by the doctrine of sympathy, nor can it be better understood by considering the general stimulus applied, or that which is applied to the extremities of the vena portarum in the intestinal canal.—By further anatomical research it seems highly probable, that some of the absorbents of the stomach or intestines, or of both, will be found always to pass through the liver; and that that peculiar state of the liver, called a schirrus, is always the consequence of drinking alkohol freely, from the inflammatory, or at least peculiar, stimulus of the alkohol, when absorbed on the absorbent vessel itself; and that it is owing to the very large proportion of absorbent vessels in the liver, that this viscus is so constantly the seat of disease in hard drinkers.

"The operation of mercury on the stomach and ductus communis choledochus, is at least of equal importance in schirrous liver, as in the state of disease the treatment of which has been considered (§ 91) and mercurial purgatives must frequently be employed:—when any benefit is to be expected from the absorption or continued action of mercury, unguentum hydrargyri fortius of the London Pharmacopœia, consisting of equal parts of quicksilver and lard, must be rubbed nightly on the epigastric and hypochondriac regions in moderate quantity, or repeated doses of mercury are to be taken internally, and, if salivation ensue, it must be discontinued only till the mouth be well."

